# Projected Impact of LDL-C and HbA_1c_ Control on Cardiovascular Outcomes in ASCVD and Diabetes

**DOI:** 10.1016/j.jacadv.2026.103308

**Published:** 2026-10-07

**Authors:** Hridhay Karthikeyan, Wenjun Fan, Ran Jin, Nafeesa Dhalwani, Lisa Head, Nathan D. Wong

**Affiliations:** a Mary and Steve Wen Cardiovascular Division, University of California-Irvine School of Medicine, Irvine, California, USA; b Center for Global Cardiometabolic Health and Nutrition, University of California-Irvine, Irvine, California, USA; c Amgen Inc, Thousand Oaks, California, USA

**Keywords:** atherosclerotic cardiovascular disease, diabetes mellitus, HbA_1c_, low-density lipoprotein cholesterol, obesity

## Abstract

**Background:**

U.S. population estimates of atherosclerotic cardiovascular disease (ASCVD) risk reduction from comprehensive cardiometabolic risk factor control remain limited among patients with ASCVD and diabetes mellitus (DM).

**Objectives:**

The objective of the study was to predict 3-year reduction in composite ASCVD event risk and number of preventable events associated with achieving guideline-based targets for low-density lipoprotein cholesterol (LDL-C) and/or hemoglobin A1c (HbA_1c_) in adults with ASCVD and DM. Among those with body mass index (BMI) ≥27 kg/m^2^, we additionally examined the impact of reducing BMI to 25 kg/m^2^.

**Methods:**

Using National Health and Nutrition Examination Survey 2011 to 2020 data weighted to the U.S. population, we identified adults with ASCVD and DM with uncontrolled LDL-C (≥70 mg/dL) and HbA_1c_ (≥7.0%). Three-year ASCVD risk was calculated using the TIMI risk score. Absolute risk was first estimated with patients’ baseline LDL-C, HbA_1c_, and BMI values, then recalculated under scenarios achieving guideline-recommended targets (70 or 55 mg/dL, 7%, and 25 kg/m^2^, respectively). Predicted relative risk reductions and predicted preventable events over 3 years were calculated.

**Results:**

We included 131 participants, representing 1.73 million U.S. adults. Predicted 3-year relative risk reductions were 18.0% and 23.7%, corresponding to 38,200 and 50,100 predicted preventable events, with LDL-C lowering to 70 mg/dL or 55 mg/dL, respectively; 29.3%, corresponding to 62,100 events, with additional control of HbA1c; and 37.0%, corresponding to 66,500 events, with additional BMI reductions.

**Conclusions:**

In U.S. adults with ASCVD and DM, LDL-C control provides the greatest projected reduction in ASCVD risk among individual risk factors, whereas comprehensive control of LDL-C, glycemia, and weight produces the greatest overall projected benefit.

It is well established that most patients with atherosclerotic cardiovascular disease (ASCVD) and diabetes mellitus (DM) are at a very high risk for future ASCVD events and mortality.^1^ Among patients at very high risk, defined as having 2 or more major ASCVD events or 1 major event accompanied by multiple high-risk conditions such as DM, subsequent ASCVD event rates are approximately 3-fold higher than in those not meeting very high-risk criteria.^2^ Despite widespread statin treatment to lower low-density lipoprotein cholesterol (LDL-C), substantial residual risk persists in this population. We have previously shown that over a median follow-up of 3.3 years, ASCVD event rates among statin-treated patients with established ASCVD were 26.9% in those with DM compared with 19.0% in those without DM, with a substantial proportion in both groups experiencing multiple events.^3^

Optimal control of LDL-C and other modifiable risk factors is therefore essential to mitigate residual ASCVD risk in patients with ASCVD and DM. However, fewer than one-fifth of patients with DM, regardless of ASCVD status, have adequately controlled key risk factors such as LDL-C, blood pressure, hemoglobin A1c (HbA_1c_), and body mass index (BMI).^4^^,^^5^ Achievement of LDL-C, blood pressure, and HbA_1c_ thresholds in those with DM is associated with a 60% lower risk of coronary heart disease, underscoring the value of composite risk factor control.^6^ Data from randomized trials have shown the benefit of composite risk factor control on reducing the risk of ASCVD events in those with ASCVD and DM.^7^

Despite this evidence, U.S. population-level projections specifically focused on adults with both ASCVD and DM that quantify: 1) the incremental benefit of LDL-C management; and 2) the additional benefit of LDL-C control when combined with simultaneous optimization of other risk factors remain limited. In this analysis, we estimate predicted ASCVD risk reductions and predicted preventable myocardial infarction (MI) and ischemic stroke (IS) events that would result from achieving guideline-recommended LDL-C, HbA_1c_, and BMI levels in adults in the United States with ASCVD and DM.

## Methods

We conducted a statistical modeling and projection study in a retrospective cohort of patients from the Unites States with ASCVD and DM using the National Health and Nutrition Examination Survey (NHANES) 2011 to 2020, which describes one of the largest and most comprehensive nationally representative cohorts of adults in the United States and comprises medical history, laboratory results, and medication information. Survey data are collected by questionnaire, including self-reported medical history of comorbidities and prescription data, and blood and urine samples are provided for laboratory measures. LDL-C values were calculated using the Friedwald equation. All NHANES participants provided informed consent.^8^ This analysis used deidentified, publicly available data and therefore was exempt from Institutional Review Board approval.

Patients in this analysis were aged ≥18 years with established ASCVD and DM and suboptimal risk factor control, defined as LDL-C ≥70 mg/dL and HbA_1c_ ≥7.0%. ASCVD was defined as a prior history of self-reported MI, coronary heart disease, or stroke. DM was defined according to NHANES criteria, including self-reported physician diagnosis, HbA_1c_ ≥6.5%, fasting glucose ≥126 mg/dL or nonfasting glucose ≥200 mg/dL, or use of insulin or oral glucose-lowering medications.^4^ A subgroup of patients who were overweight or obese (BMI ≥27 kg/m^2^) was additionally evaluated for risk reduction associated with BMI management. To obtain projections of the eligible sample (and predicted preventable events), NHANES uses a complex, multistage, stratified probability sampling design to obtain a nationally representative sample of the civilian, noninstitutionalized U.S. population.^8^ Sample weights were applied to account for differences in any unequal selection probabilities, oversampling, nonresponse, and poststratification adjustments. Analyses incorporated NHANES sampling weights, strata, and primary sampling units according to National Center for Health Statistics analytic guidelines.^8^ Final weights were created by dividing the 2-year weights by the number of cycles combined (9.3 years in this study). The 3-year risk reduction in a composite outcome of nonfatal and/or fatal MI and IS was predicted under scenarios in which patients achieved guideline-recommended levels for LDL-C and/or HbA_1c_. Analyses were conducted using 2 recommended LDL-C levels: 70 mg/dL, consistent with the threshold for addition of nonstatin therapy from the 2018 American Heart Association/American College of Cardiology/Multi-Society Guideline for patients with very high-risk ASCVD,^9^ and 55 mg/dL, the more contemporary recommendation from the 2026 American College of Cardiology/American Heart Association/Multi-Society Dyslipidemia Guideline for patients with very high-risk ASCVD, including those who have diabetes.^10^ The HbA_1c_ target of 7% was based on the American Diabetes Association standards of care.^11^ In patients who were additionally overweight or obese (BMI ≥27 kg/m^2^), cardiovascular risk reduction was further predicted with achievement of LDL-C, HbA_1c_, and BMI targets individually and in combination. A BMI target of 25 kg/m^2^ was selected to reflect nonoverweight/obese status and aligned with American Diabetes Association recommendations for weight management in patients with BMI ≥27 kg/m^2^ and comorbidities (such as ASCVD in the patients in this study).^12^

The TIMI Risk Score for Atherothrombosis in Diabetes (TIMI-TRS) is a validated model for predicting the 3-year risk of MI or IS, derived from a pooled cohort of 42,181 patients with DM enrolled in 4 TIMI clinical trials. The model incorporates prior ASCVD, LDL-C, HbA_1c_, BMI, and other patient characteristics (Supplemental Table 1).^13^ For each participant, predicted baseline 3-year risk was calculated using all available observed patient characteristics. For each hypothetical target-attainment scenario, predicted risk was recalculated for each participant after setting LDL-C to 70 mg/dL or 55 mg/dL, HbA_1c_ to 7%, and/or BMI to 25 kg/m^2^ (to replicate “post-treatment” risk estimates). Relative risk reductions (RRRs) were calculated as the difference between the mean risk at baseline (across all participants) and the mean risk with each target-attainment scenario, divided by the mean risk at baseline. To estimate the corresponding numbers of ASCVD events, the mean baseline risk and the mean risk at target attainment were individually multiplied by the NHANES-weighted population size. The difference between the resulting event counts at baseline and at target attainment represented the model-estimated reduction in MI or IS events in a 3-year period, referred to as the predicted number of preventable events.

Exploratory analyses were performed stratified by sex (female/male) and race/ethnicity (Asian, Black, Hispanic, White, and other) for the estimation of 3-year predicted ASCVD risk reduction and predicted preventable events. NHANES sample-weighting procedures as described previously were used to adjust for any oversampling of these racial/ethnic groups to ensure representativeness of their respective U.S. population proportions.

Certain TIMI-TRS variables, namely percutaneous coronary intervention, coronary artery bypass graft, and peripheral arterial disease, were unavailable in NHANES. As such, to obtain more accurate population risk estimates, proportions for percutaneous coronary intervention, coronary artery bypass graft, and peripheral arterial disease were imputed into the sample based on prevalence estimates for these 3 variables in patients with ASCVD and DM in the MarketScan U.S. multipayer claims database (January 2016-November 2024). The SAS SURVEYSELECT procedure was used to randomly select patients to achieve the closest number of patients to the estimated prevalence rates for the 3 variables, from which NHANES sample weights were applied to project our study sample to the greater U.S. population with ASCVD and DM. To assess the impact of imputation on calculated risk scores, a sensitivity analysis was conducted in which these conditions were conservatively assumed to be absent in the study sample.

Statistics are reported as U.S. population–weighted estimates as previously described. As this was a secondary NHANES analysis designed to estimate predicted cardiovascular risk rather than test a prespecified hypothesis, no a priori sample size or power calculation was performed; 95% CIs were calculated using the SURVEYMEANS procedure in SAS, which accounts for the weighted frequencies of the participants.

Data analyses were performed using SAS (version 9.4, SAS Institute). Data are available from NHANES.^8^ No additional data are available from the authors. Data collection was conducted in accordance with the ethical principles that have their origin in the Declaration of Helsinki and was consistent with the guidelines for Good Clinical Practice.

## Results

### Baseline characteristics

From U.S. NHANES data (2011-2020), 131 patients, representing approximately 1.73 million adults in the United States, met the eligibility criteria and were included in the primary analysis. A subgroup of 111 patients, representing approximately 1.53 million adults in the United States, also had a BMI ≥27 kg/m^2^ (Figure 1).

**Figure 1:**
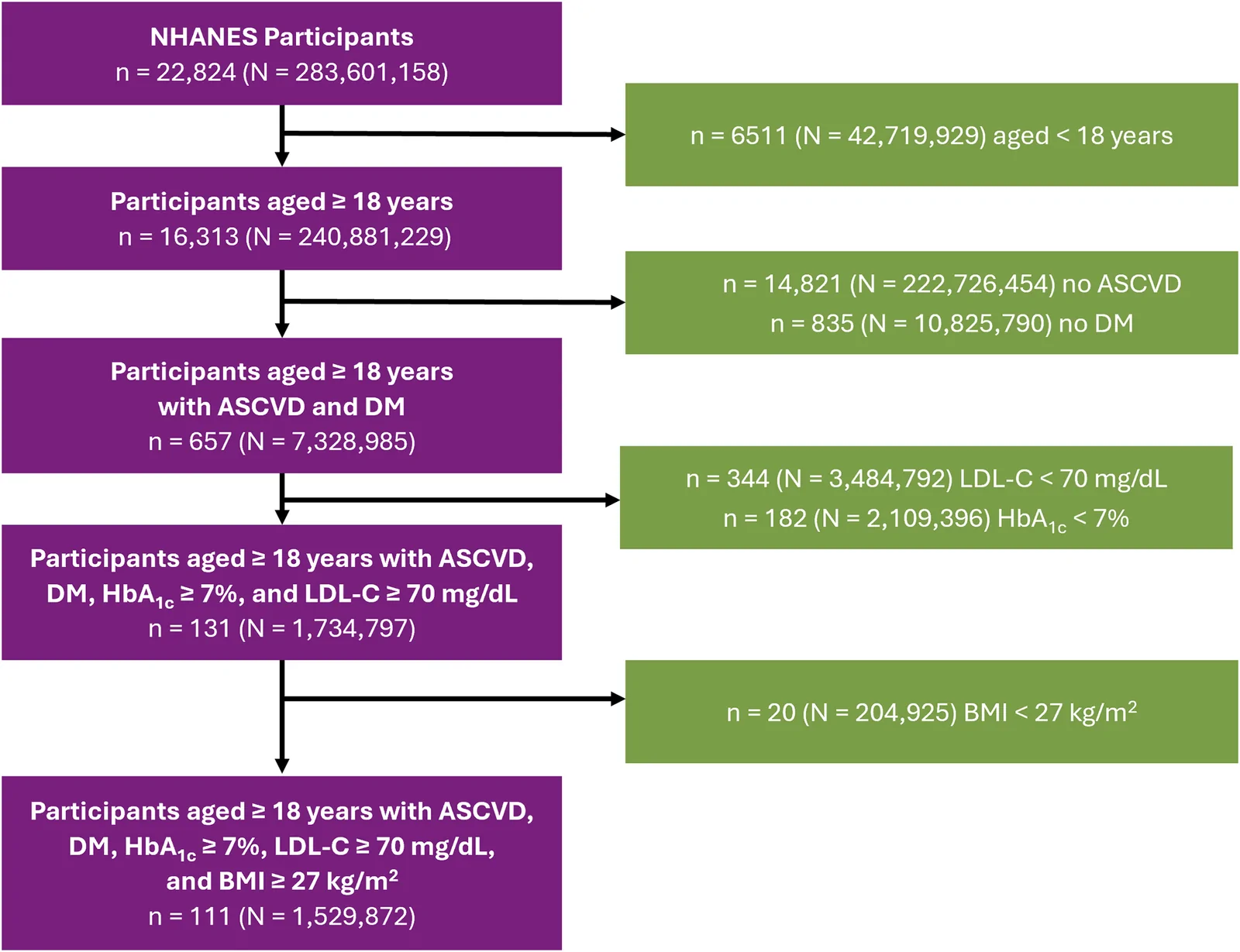
Eligible Sample Selection From NHANES (2011-2020) Participants from NHANES who were ≥18 years of age, had ASCVD with LDL-C ≥70 mg/dL, and had DM with HbA_1c_ ≥7% were included in the primary analysis. Patients in this group who also had a BMI ≥27 kg/m^2^ were included in the secondary analysis. ASCVD = atherosclerotic cardiovascular disease; BMI = body mass index; DM = diabetes mellitus; HbA_1c_ = hemoglobin A1c; LDL-C = low-density lipoprotein cholesterol; NHANES = National Health and Nutrition Examination Survey.

Descriptive characteristics of these patients are shown in Table 1. In the primary sample, the mean (95% CI) age was 63.3 (61.5-65.1) years; 69.6% were males and 69.4% were White. The mean (95% CI) systolic/diastolic blood pressure was 132.4 (128.3-136.5)/69.4 (67.2-71.4) mm Hg, and LDL-C was 110.9 (105.0-116.8) mg/dL. A history of MI was present in 44.3% of patients, and 40.9% had a prior stroke. Overall, 13.6% of patients reported current smoking. Characteristics were similar in the subgroup that also had BMI ≥27 kg/m^2^ (Table 1).

**Table 1:** Descriptive Statistics for Primary- and Secondary-Sample NHANES Patients

	Primary Sample (n = 131) 1.73 M	Secondary Sample (n = 111) 1.53 M
Age, y, mean (95% CI)	63.3 (61.5-65.1)	62.7 (61.1-64.3)
Age group, weighted percentage (frequency n [unweighted], N [weighted])		
<65 y	53.4 (64, 0.92 M)	56.8 (57, 0.87 M)
65-74 y	29.3 (41, 0.51 M)	29.3 (36, 0.45 M)
≥75 y	17.3 (26, 0.30 M)	13.9 (18, 0.21 M)
Sex, weighted percentage (frequency n [unweighted], N [weighted])		
Male	69.6 (78, 1.21 M)	68.2 (66, 1.04 M)
Female	30.4 (53, 0.52 M)	31.8 (45, 0.49 M)
Racial or ethnic group, weighted percentage (frequency n [unweighted], N [weighted])		
Mexican American or other Hispanic	10.3 (35, 0.18 M)	9.6 (30, 0.15 M)
Non-Hispanic Asian	2.5 (9, 0.04 M)	1.9 (6, 0.03 M)
Non-Hispanic Black	12.1 (34, 0.21 M)	11.3 (28, 0.17 M)
Non-Hispanic White	69.4 (47, 1.20 M)	70.7 (41, 1.08 M)
Other^a^	5.7 (6, 0.10 M)	6.5 (6, 0.10 M)
Body mass index, kg/m^2^, mean (95% CI)	34.8 (33.0-36.6)	36.1 (35.1-37.1)
Blood pressure, mm Hg, mean (95% CI)		
Systolic	132.4 (128.3-136.5)	131.8 (127.5-136.1)
Diastolic	69.4 (67.2-71.4)	69.6 (67.6-71.6)
Systolic blood pressure group, weighted percentage (frequency n [unweighted], N [weighted])		
<140 mm Hg	71.3 (86, 1.24 M)	74.0 (73, 1.13 M)
140-159 mm Hg	17.6 (26, 0.30 M)	14.5 (22, 0.22 M)
≥160 mm Hg	11.1 (19, 0.19 M)	11.5 (16, 0.18 M)
LDL-C, mg/dL, mean (95% CI)	110.9 (105.0-116.8)	111.3 (105.8-116.8)
eGFR, mL/min/1.73 m^2^, mean (95% CI)	75.3 (71.8-78.8)	76.3 (72.6-80.0)
eGFR group, weighted percentage (frequency n [unweighted], N [weighted])		
<60 mL/min/1.73 m^2^	28.8 (40, 0.50 M)	27.5 (31, 0.42 M)
≥60 mL/min/1.73 m^2^	71.2 (91, 1.23 M)	72.5 (80, 1.11 M)
HbA_1c_, %, mean (95% CI)	8.9 (8.5-9.3)	9.0 (8.6-9.4)
Insulin medication usage, weighted percentage (frequency n [unweighted], N [weighted])	48.4 (69, 0.84 M)	47.9 (56, 0.73 M)
Current smoker, weighted percentage (frequency n [unweighted], N [weighted])	13.6 (16, 0.24 M)	14.3 (13, 0.22 M)
UACR, mg/g, mean (95% CI)	411.0 (181.1-640.9)	416.4 (165.1-667.7)
UACR group, weighted percentage (frequency n [unweighted], N [weighted])		
<30 mg/g	51.4 (61, 0.89 M)	50.3 (52, 0.77 M)
30-299 mg/g	24.4 (34, 0.42 M)	25.5 (29, 0.39 M)
≥300 mg/g	24.2 (36, 0.42 M)	24.2 (30, 0.37 M)
ASCVD history, weighted percentage (frequency n [unweighted], N [weighted])		
Coronary artery disease	45.2 (56, 0.78 M)	46.0 (49, 0.70 M)
MI	44.3 (64, 0.77 M)	43.8 (52, 0.67 M)
Stroke	40.9 (47, 0.71 M)	41.1 (41, 0.63 M)
PAD^b^	7.6 (10, 0.13 M)	7.2 (8, 0.11 M)
PCI^b^	20.6 (27, 0.36 M)	20.7 (23, 0.32 M)
CABG^b^	16.0 (21, 0.28 M)	16.2 (18, 0.25 M)

ASCVD = atherosclerotic cardiovascular disease; CABG = coronary artery bypass graft; eGFR = estimated glomerular filtration rate; HbA_1c_ = hemoglobin A1c; LDL-C = low-density lipoprotein cholesterol; M = million; MI = myocardial infarction; NHANES = National Health and Nutrition Examination Survey; PAD = peripheral arterial disease; PCI = percutaneous coronary intervention; UACR = urine albumin-creatinine ratio.

a Other includes Asian and race/ethnicity not specified.

b Proportions for PCI, CABG, and PAD were imputed into the sample based on MarketScan prevalence estimates for those 3 variables in patients with diabetes and ASCVD.

### Changes in 3-year ASCVD risk and predicted preventable MI or IS events

Reducing LDL-C to the guideline-recommended level of 70 mg/dL or 55 mg/dL was predicted to have an RRR (95% CI) of 18.0% (17.2%-18.8%) and 23.7% (22.9%-24.5%), respectively. Achieving an HbA_1c_ of 7% alone had a more modest predicted risk reduction (RRR: 7.8% [6.0%-9.6%]). Simultaneous control of both LDL-C and HbA_1c_ lowered the predicted risk by 24.1% (22.6%-25.6%) when LDL-C was reduced to 70 mg/dL and by 29.3% (27.8%-30.9%) when LDL-C was reduced to 55 mg/dL (Figure 2). At the population level, LDL-C control to 70 mg/dL was predicted to prevent approximately 38,200 (95% CI: 34,700-41,500) MI or IS events over 3 years, and LDL-C control to 55 mg/dL was predicted to prevent 50,100 (45,600-54,300) events. HbA_1c_ control to 7% was predicted to prevent approximately 16,500 (14,600-18,200) events over 3 years. Combined control of LDL-C and HbA_1c_ was projected to prevent approximately 51,000 (46,500-55,300) and 62,100 (56,700-67,300) events for LDL-C targets of 70 mg/dL and 55 mg/dL, respectively (Figure 2).

**Figure 2:**
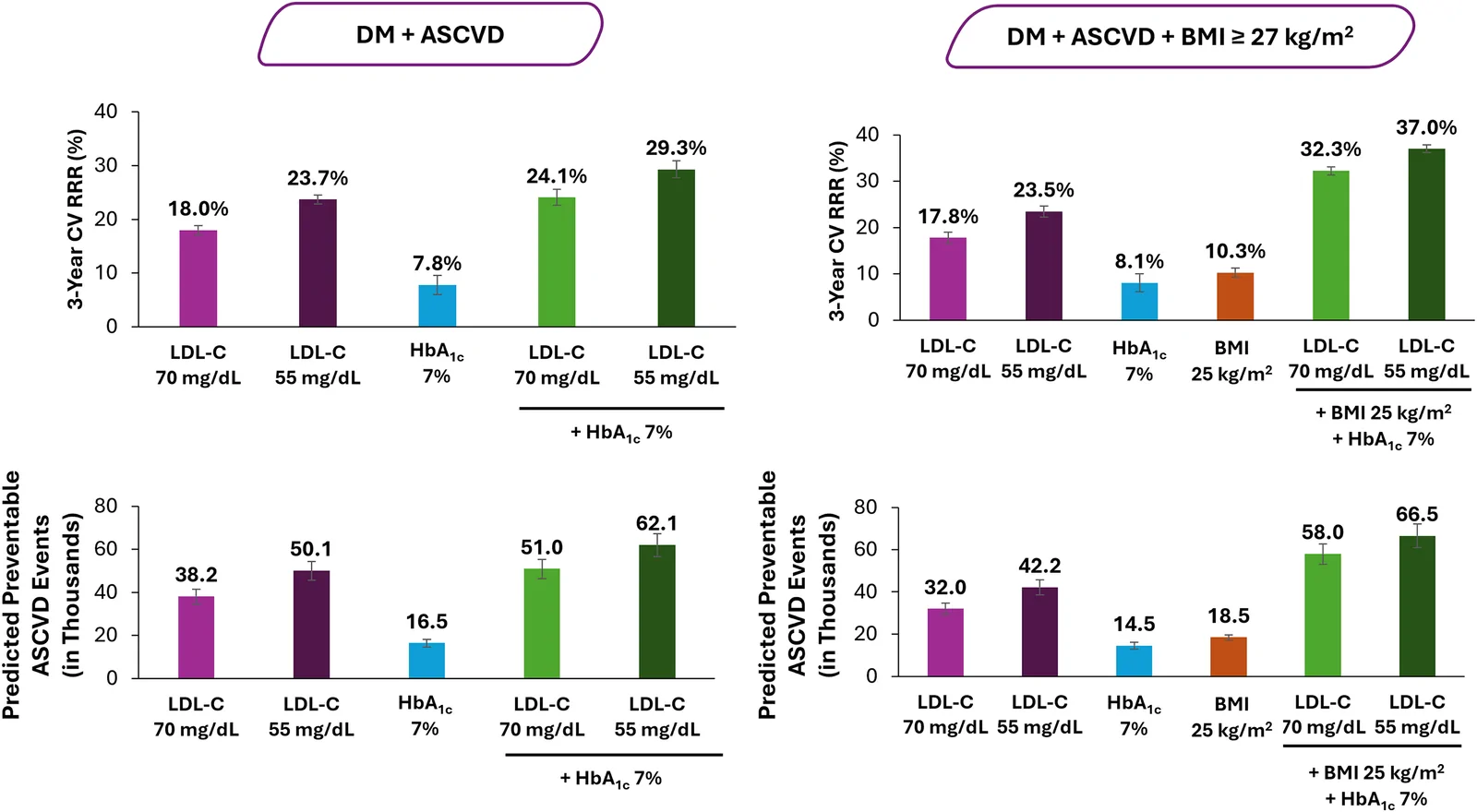
Risk Factor Management and 3-Year ASCVD RRR and Preventable Events The 3-year CV RRR was predicted for control of LDL-C and HbA_1c_ (individually and in combination) in patients with ASCVD and DM and was translated to total predicted preventable ASCVD events in the U.S. population. This analysis was repeated in patients who also had BMI ≥27 kg/m^2^, with individual and combined control of LDL-C, HbA_1c_, and BMI. CV risk is defined as risk of the composite endpoint of fatal/nonfatal myocardial infarction and stroke. Error bars represent 95% CIs. CV = cardiovascular; RRR = relative risk reduction; other abbreviations as in Figure 1.

Among the subgroup of patients with BMI ≥27 kg/m^2^, additional risk reductions were predicted by applying a BMI target of 25 kg/m^2^ (Figure 2). Consistent with findings in the primary analysis, LDL-C control in this subgroup achieved the greatest RRRs (17.8% [95% CI: 16.6%-19.0%] and 23.5% [22.3%-24.7%] for LDL-C 70 mg/dL and 55 mg/dL, respectively), compared with control of HbA_1c_ to 7% (RRR: 8.1% [6.2%-10.0%]) or BMI to 25 kg/m^2^ (RRR, 10.3% [9.3%-11.3%]). Simultaneously controlling LDL-C to 70 mg/dL, HbA_1c_ to 7%, and BMI to 25 kg/m^2^ corresponded to an RRR of 32.3% (31.4%-33.1%), which increased to 37.0% (36.2%-37.9%) when LDL-C was reduced to 55 mg/dL. At the population level, LDL-C control alone to 70 mg/dL or 55 mg/dL was projected to prevent approximately 32,000 (95% CI: 29,200-34,700) or 42,200 (38,600-45,700) events, respectively, over 3 years. Individual control of HbA_1c_ to 7% was projected to prevent approximately 14,500 (12,900-16,100) events, and a BMI reduction to 25 kg/m^2^ alone was projected to prevent approximately 18,500 (17,100-19,700) events. Simultaneous control of all 3 risk factors was projected to prevent approximately 58,000 (53,100-62,700) MI or IS events with an LDL-C threshold of 70 mg/dL and approximately 66,500 (60,900-72,100) events with an LDL-C threshold of 55 mg/dL (Central Illustration).

**Central Illustration:**
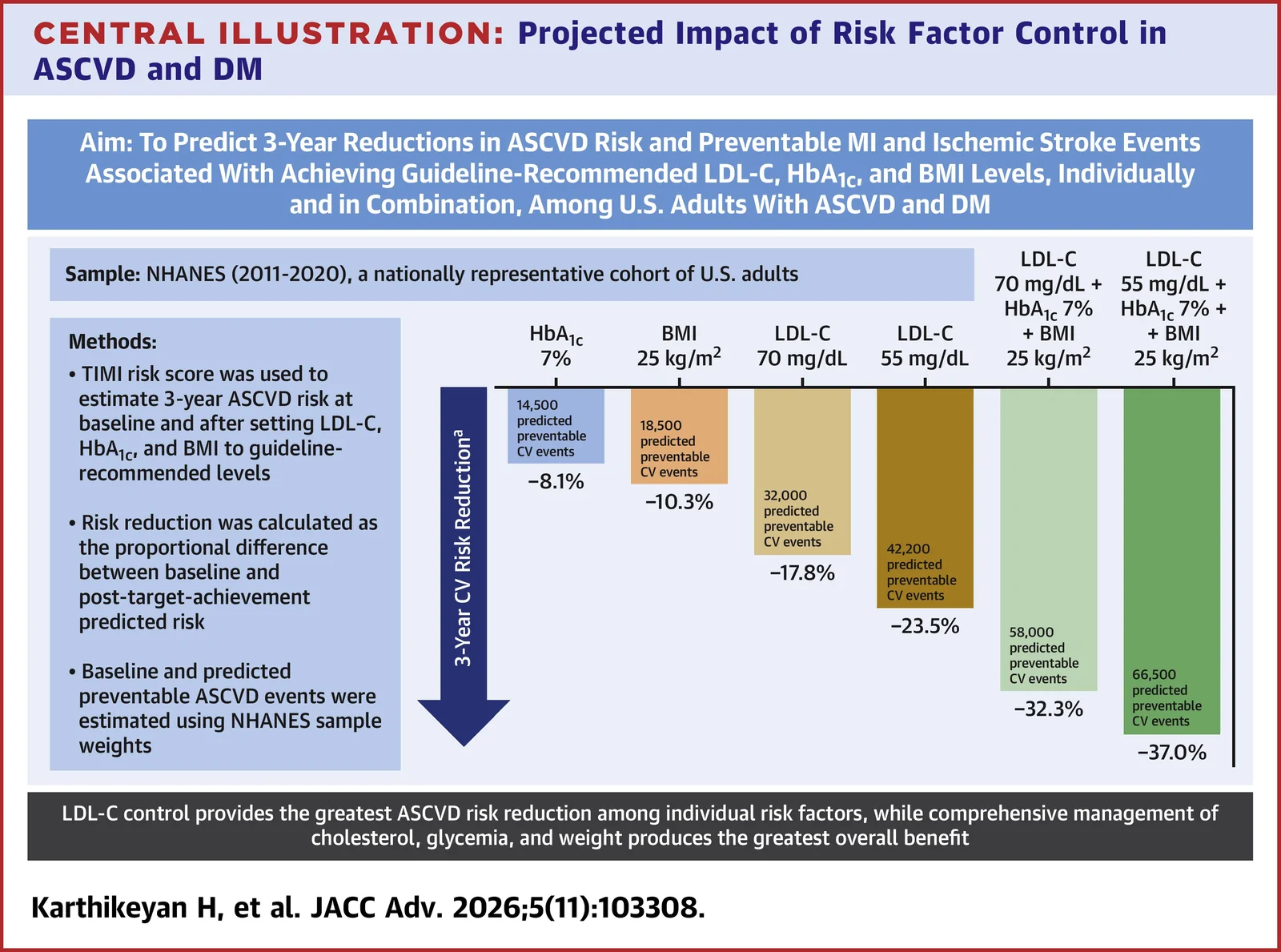
Projected Impact of Risk Factor Control in ASCVD and DM ^a^In patients with uncontrolled risk factors (LDL-C ≥70 mg/dL, HbA_1c_ ≥7.0%, BMI ≥27 kg/m^2^). In a model of risk factor control that included LDL-C, HbA_1c_, and BMI, the greatest predicted decrease in CV risk and CV events was seen with simultaneous control of all 3 risk factors. Individually, LDL-C control had the greatest predicted effect on CV risk. ASCVD = atherosclerotic cardiovascular disease; BMI = body mass index; CV = cardiovascular; DM = diabetes mellitus; HbA_1c_ = hemoglobin A_1c_; LDL-C = low-density lipoprotein cholesterol; MI = myocardial infarction; NHANES = National Health and Nutrition Examination Survey.

### Stratified analysis by sex and race/ethnicity

Across sex and race/ethnicity subgroups, combined risk factor control was consistently associated with greater predicted 3-year cardiovascular risk reduction and more predicted preventable events than individual risk factor control alone (Supplemental Figures 1-4). For example, among male adults, adding BMI control to LDL-C and HbA_1c_ control was associated with an additional predicted benefit, with the largest RRR (95% CI) observed being 38.9% (38.0%-39.8%) under the scenario combining intensive LDL-C lowering to 55 mg/dL, HbA_1c_ control to 7%, and BMI reduction to 25 kg/m^2^, compared with RRRs observed for individual risk factor control alone ranging from 9.4% (7.0%-11.9%) to 24.4% (23.2%-25.5%) (Supplemental Figures 1 to 4).

The absolute number of predicted preventable events appeared to be greater in males than in females and greatest among White patients (Supplemental Figures 2 and 4 and 4), primarily reflecting the larger representation of these groups in the study population.

## Discussion

Our study provides the first U.S. population–level projections of ASCVD risk reduction benefits associated with composite risk factor control in patients with coexisting ASCVD, DM, and overweight or obesity, incorporating contemporary LDL-C treatment targets and using a validated risk model. In this U.S. population–based analysis of adults with ASCVD and DM, we reported that achieving guideline-recommended thresholds for LDL-C, HbA_1c_, and BMI was predicted to substantially reduce the risk of MI and IS events over a 3-year period. LDL-C control alone was associated with the largest predicted risk reduction compared to individual control of HbA_1c_ or BMI, with additional incremental benefits observed with concurrent glycemic and weight control on top of LDL-C control. At the population level, this triple risk factor management approach was projected to prevent up to 66,500 MI or IS events over 3 years. Risk reduction patterns were generally consistent across sex and race/ethnicity subgroups, with combined risk factor control associated with the greatest predicted benefit in all evaluated groups. These findings highlight the central role of LDL-C control in managing residual ASCVD risk in this high-risk population, while underscoring the added value of addressing multiple cardiometabolic risk factors simultaneously.

Despite strong evidence supporting comprehensive risk factor management, control of key cardiometabolic risk factors among adults in the United States with ASCVD and DM remains suboptimal. In our prior work among adults in the United States with DM and prior ASCVD, although 73.3%, 26.4%, and 32.9% met recommended thresholds for HbA_1c_ (<7.0%), LDL-C (<70 mg/dL), and BMI (<30 kg/m^2^), respectively, only 2.7% were simultaneously at target for all 5 risk factors, including HbA_1c_, LDL-C, BMI, blood pressure, and nonsmoking status.^6^ In a pooled analysis of adults in the United States with DM without established ASCVD, achievement of combined treatment targets for blood pressure, LDL-C, and HbA_1c_ was associated with a 60% lower risk of coronary heart disease, with LDL-C control contributing the largest individual risk reduction for coronary heart disease events (41%).^6^ These observations are consistent with the findings of the current study, which demonstrate that LDL-C control contributes the greatest reduction in ASCVD risk among patients with ASCVD and DM. Further supporting this concept, the BARI-2D trial showed that patients with DM and stable coronary heart disease with fewer risk factors (0-2) in control during follow-up experienced a 2-fold increase in risk of death and a 1.7-fold increase in risk of major cardiovascular events compared to those with more risk factors (6) in control.^7^ Together, these data reinforce the importance of prioritizing LDL-C lowering while simultaneously addressing other modifiable risk factors to reduce residual ASCVD risk.

### Strengths and limitations

Our study has several strengths and limitations. A key strength of this study is the use of NHANES, which is the only nationally representative data set that incorporates sample weighting, enabling estimation of preventable ASCVD events at the U.S. population level. In addition, we leveraged a validated, DM-specific risk model developed and externally validated in more than 42,000 individuals with DM from contemporary clinical trial populations, including a large proportion with established ASCVD.^13^ This model demonstrates good discrimination and calibration for predicting MI or IS and is well aligned with the population studied. Several limitations should be acknowledged. First, although NHANES enables population-level extrapolation, the number of eligible patients based on the inclusion criteria for our analytic sample was relatively small, which may limit the precision of estimates, particularly in analyses stratified by sex and race/ethnicity. These stratified analyses were exploratory and should be interpreted cautiously. Second, we simulated control of LDL-C, HbA_1c_, and BMI to uniform thresholds across all patients not at the recommended level. However, the limitations in our sample size precluded looking at control of other factors, such as blood pressure or smoking, in addition to our prespecified parameters for LDL-C, HbA_1c_, and BMI control. In clinical practice, responses to pharmacologic and lifestyle interventions are heterogeneous, and attainment of targets may vary based on baseline levels and treatment intensity, potentially leading to overestimation or underestimation of risk reductions for some individuals. Moreover, our statistical modeling study estimated cardiovascular risk reduction using the TIMI-TRS equation based on risks calculated from controlled levels of LDL-C, HbA_1c_, and/or BMI rather than the effects of actually achieving these levels from specific therapies, which can also vary depending on the degree of adherence and persistence in using such therapies. As a result, the model does not account for potential pleiotropic cardiovascular benefits of interventions (eg, statins or glucagon-like peptide-1 receptor agonists) that may extend beyond their effects on these biomarkers. Moreover, the TIMI-TRS equations, the only available equations with our key variables of interest (LDL-C, HbA_1c_, and BMI) for use in secondary prevention samples with diabetes, were developed from clinical trial samples and have limited validity in population-based samples such as those from NHANES. Third, ASCVD history in NHANES is based on self-reporting, which may be less precise than adjudicated diagnoses derived from administrative or clinical data sources (e.g., International Classification of Disease codes). Another important limitation inherent in the use of NHANES is that the sample weighting procedures used to extrapolate data from an individual participant to a larger sample may not fully account for differences in both known and unknown factors between the individual and the larger sample they represent. Finally, several components of the TIMI-TRS, including percutaneous coronary intervention, coronary artery bypass graft, and peripheral arterial disease, were not available in NHANES and were imputed using prevalence estimates from the MarketScan multipayer claims database. Although this imputation may not accurately estimate risk in a given individual, it would provide more accurate estimates of absolute ASCVD risk at the population level. However, sensitivity analyses assuming the absence of these conditions yielded similar RRR estimates, supporting the robustness of our findings (Supplemental Table 2).

## Conclusions

Among patients with ASCVD and DM, LDL-C control is predicted to confer the greatest risk reduction in recurrent MI and IS events, with larger benefits observed at an LDL-C target of 55 mg/dL compared to 70 mg/dL. Additional control of HbA_1c_ and BMI provides further clinically meaningful reduction in risk and preventable events. These findings emphasize the importance of comprehensive cardiometabolic risk factor management, anchored by aggressive LDL-C lowering, to address the substantial residual ASCVD risks in this high-risk population.

Perspectives **COMPETENCY IN MEDICAL KNOWLEDGE:** It is well established that controlling LDL-C, HbA_1c_, and BMI reduces the risk of adverse cardiovascular events. Here, we have quantified the extent to which each risk factor individually contributes to risk and predicted the number of cardiovascular events that could be prevented over the course of 3 years with better risk factor control.

**TRANSLATIONAL OUTLOOK:** Current lipid guidelines are evolving to recommend lower LDL-C levels for patients at varying levels of cardiovascular risk. However, it is up to clinicians to test LDL-C levels and to manage lipid-lowering therapy where needed. Our results should encourage clinicians to manage LDL-C, HbA_1c_, and BMI to the greatest extent possible in their patients.

## Funding support and author disclosures

This study was supported by a contract from Amgen Inc, with the University of California-Irvine. Dr Wong has received research support through his institution from Amgen Inc, Novartis, and Lilly (through UC-San Diego); has received consultant fees from Heart Lung AI, Lilly, and Novartis. Drs Jin, Dhalwani, and Head are employees and shareholders of Amgen Inc. All other authors have reported that they have no relationships relevant to the contents of this paper to disclose.
