# Supplementary material for: Projected Impact of LDL-C and HbA1c Control on Cardiovascular Outcomes in ASCVD and Diabetes
Source: JACC Adv. 2026 Oct 7;5(11):103308. doi: 10.1016/j.jacadv.2026.103308 (PMC13676218; doi:10.1016/j.jacadv.2026.103308)
Supplement: Supplementary file 1 [file mmc1.pdf]

SUPPLEMENTAL APPENDIX

Table of Contents

SUPPLEMENTAL TABLE 1 Coefficients and Equations From the TIMI-TRS. 1

SUPPLEMENTAL TABLE 2 Sensitivity Analyses of Estimated 3-Year ASCVD Risk in Patients With DM, ASCVD, HbA_{1c} ≥ 7%, and LDL-C ≥ 70 mg/dL (With or Without BMI ≥ 27 kg/m^2), Assuming Absence of PAD, PCI, and CABG. 2

SUPPLEMENTAL FIGURE 1 Predicted 3-Year ASCVD RRR With Risk Factor Control by Sex and Race/Ethnicity 3

SUPPLEMENTAL FIGURE 2 Predicted Preventable CV Events With Risk Factor Control by Sex and Race/Ethnicity 4

SUPPLEMENTAL FIGURE 3 Predicted 3-Year ASCVD RRR With Added BMI Control by Sex and Race/Ethnicity 5

SUPPLEMENTAL FIGURE 4 Predicted Preventable CV Events With Added BMI Control by Sex and Race/Ethnicity 6

SUPPLEMENTAL TABLE 1 Coefficients and Equations From the TIMI-TRS.

**Parameter**	**Beta Coefficient**
**Age**	
65–74 years	0.263
≥ 75 years	0.539
**Sex, male**	0.285
**Body mass index**	0.0108
**LDL-C**	0.0051
**SBP**	
140–159 mm Hg	0.141
≥ 160 mm Hg	0.406
**Tobacco use**	0.318
**Insulin use**	0.262
**HbA_{1c}**	0.041
**eGFR < 60 mL/min/1.73 m^2**	0.202
**UACR**	
30–< 300 mg/g	0.233
≥ 300 mg/g	0.451
**Coronary artery disease**	0.474
**Prior MI**	0.287
**Prior PCI**	0.397
**Prior CABG**	0.293
**Ischemic stroke**	0.696
**PAD**	0.270

Adapted from Berg et al (*J Am Coll Cardiol.* 2023;81[25]:2391-2402.

https://doi.org/10.1016/j.jacc.2023.04.031).

CABG, coronary artery bypass graft; eGFR, estimated glomerular filtration rate;

HbA_{1c}, hemoglobin A_{1c}; LDL-C, low-density lipoprotein cholesterol; MI, myocardial infarction;

PAD, peripheral arterial disease; PCI, percutaneous coronary intervention;

SBP, systolic blood pressure; TIMI-TRS, TIMI Risk Score for Atherothrombosis in Diabetes;

UACR, urine albumin-creatinine ratio.

SUPPLEMENTAL TABLE 2 Sensitivity Analyses of Estimated 3-Year ASCVD Risk in Patients With DM, ASCVD, $HbA_{1c} \geq 7\%$, and LDL-C ≥ 70 mg/dL (With or Without BMI ≥ 27 kg/m^2), Assuming Absence of PAD, PCI, and CABG.

	**Relative Risk Reduction, % (95% CI)**	**Predicted Preventable Events (95% CI)[a]**
**Primary sample, n = 131 (1.73M)**		
LDL-C 70 mg/dL	18.4 (17.8, 19.0)	33.0 (30.0, 35.7)
LDL-C 55 mg/dL	24.1 (23.5, 24.7)	43.2 (39.4, 46.7)
HbA_{1c} 7%	8.0 (6.0, 10.0)	14.4 (12.7, 16.1)
LDL-C 70 mg/dL + HbA_{1c} 7%	24.6 (20.8, 28.4)	44.1 (40.1, 47.9)
LDL-C 55 mg/dL + HbA_{1c} 7%	29.9 (26.4, 33.4)	53.6 (48.9, 58.1)
**Secondary sample, n = 111 (1.53M)**		
LDL-C 70 mg/dL	18.4 (17.7, 19.1)	28.9 (26.0, 32.0)
LDL-C 55 mg/dL	24.1 (23.5, 24.8)	37.9 (34.3, 41.6)
HbA_{1c} 7%	8.3 (6.2, 10.3)	13.0 (11.5, 14.8)
BMI 25 kg/m^2	10.1 (8.8, 11.4)	15.9 (15.1, 16.7)
LDL-C 70 mg/dL + HbA_{1c} 7% + BMI 25 kg/m^2	32.9 (32.5, 33.3)	51.7 (47.0, 56.5)
LDL-C 55 mg/dL + HbA_{1c} 7% + BMI 25 kg/m^2	37.5 (37.0, 38.1)	59.1 (53.9, 64.4)

All risk scores were computed using the TIMI-TRS. Values may not add up to the total due to rounding errors.

[a]Weighted frequency values are listed in thousands.

ASCVD, atherosclerotic cardiovascular disease; BMI, body mass index; CABG, coronary artery bypass graft; CI, confidence interval; DM, diabetes mellitus; HbA_{1c}, hemoglobin A_{1c}; LDL-C, low-density lipoprotein cholesterol; M, million; PAD, peripheral arterial disease; PCI, percutaneous coronary intervention; TIMI-TRS, TIMI Risk Score for Atherothrombosis in Diabetes.

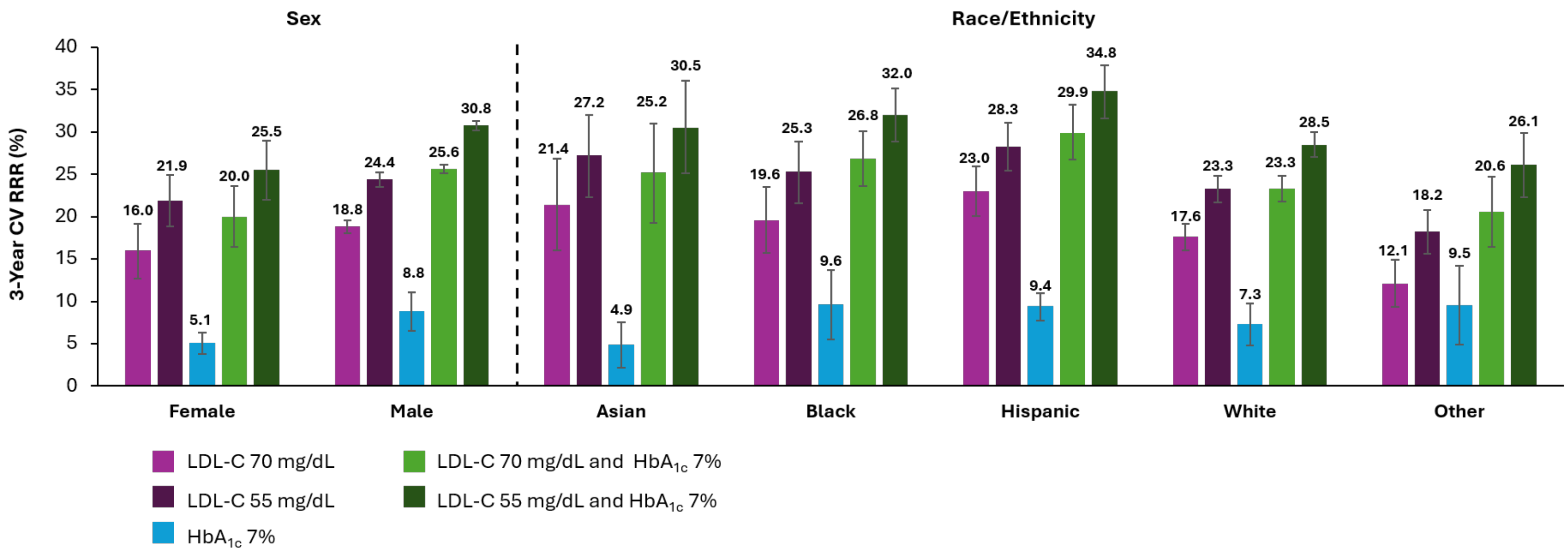

SUPPLEMENTAL FIGURE 1 Predicted 3-Year ASCVD RRR With Risk Factor Control by Sex and Race/Ethnicity

The 3-year ASCVD RRR was predicted for LDL-C and HbA_{1c} control (individually and combined) when the cohort with ASCVD, DM, LDL-C $\geq$ 70 mg/dL, and $HbA_{1c} \geq 7\%$ was divided by sex and by race/ethnicity. All risk scores were computed using the TIMI risk score algorithm. Error bars represent 95% CIs. ASCVD, atherosclerotic cardiovascular disease; CI, confidence interval; CV, cardiovascular; DM, diabetes mellitus; HbA_{1c}, hemoglobin A_{1c}; LDL-C, low-density lipoprotein cholesterol; RRR, relative risk reduction.

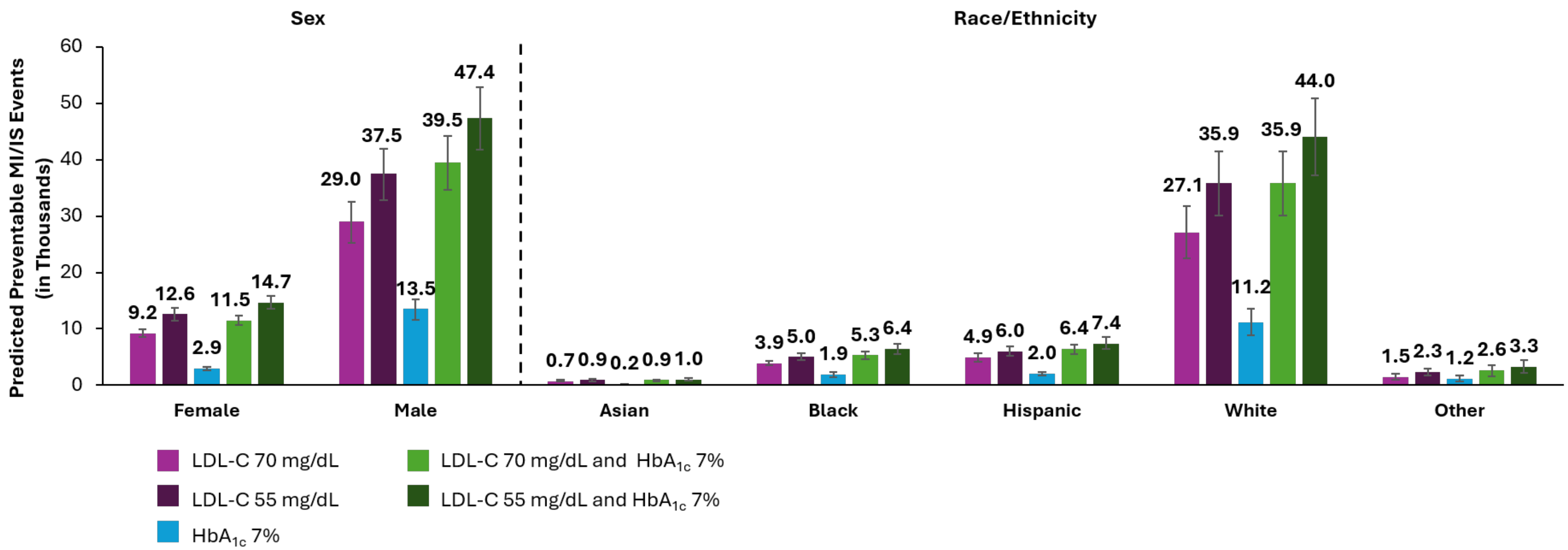

SUPPLEMENTAL FIGURE 2 Predicted Preventable CV Events With Risk Factor Control by Sex and Race/Ethnicity

The number of predicted preventable MI and IS events over the course of 3 years that would result from control of LDL-C and HbA_{1c} (individually and in combination) was estimated in the cohort with ASCVD, DM, LDL-C ≥ 70 mg/dL, and HbA_{1c} ≥ 7%, stratified by sex and by race/ethnicity. All risk scores were computed using the TIMI risk score algorithm. Error bars represent 95% CIs.

ASCVD, atherosclerotic cardiovascular disease; CI, confidence interval; CV, cardiovascular; DM, diabetes mellitus; HbA_{1c}, hemoglobin A_{1c}; IS, ischemic stroke; LDL-C, low-density lipoprotein cholesterol; MI, myocardial infarction.

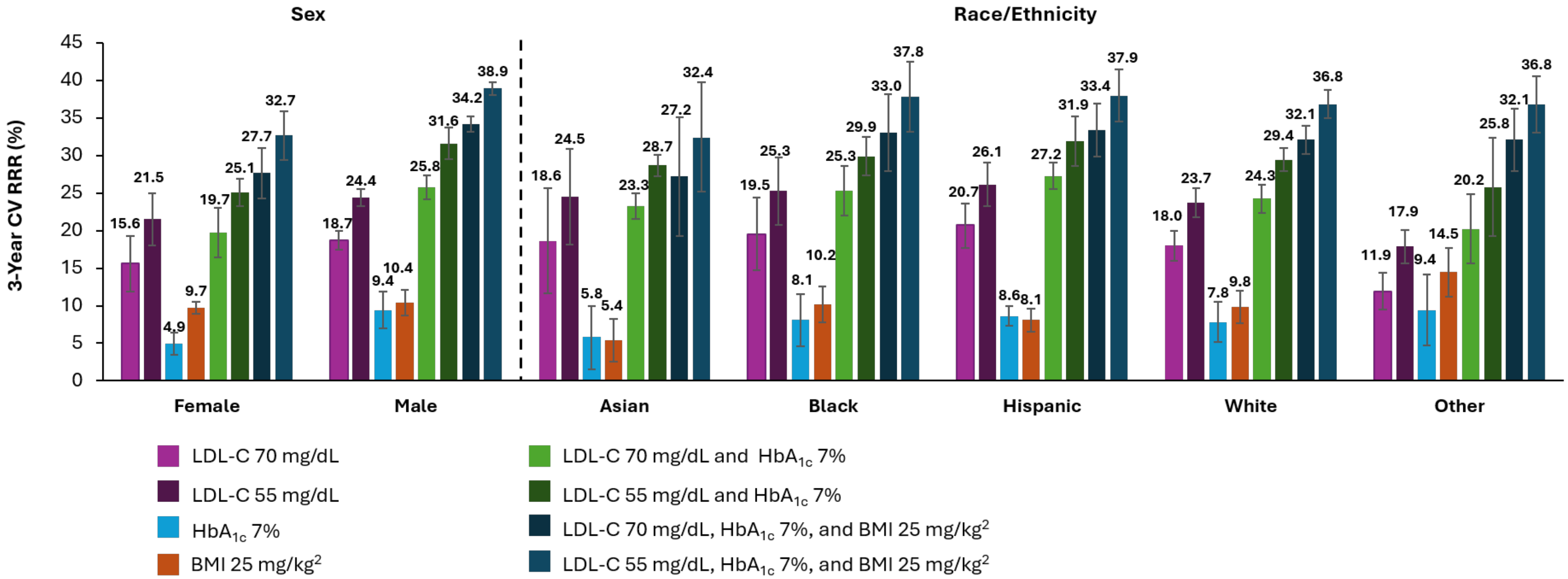

SUPPLEMENTAL FIGURE 3 Predicted 3-Year ASCVD RRR With Added BMI Control by Sex and Race/Ethnicity

The 3-year ASCVD RRR was predicted for LDL-C, HbA_{1c}, and BMI control (individually and combined) when the cohort with ASCVD, DM, LDL-C ≥ 70 mg/dL, HbA_{1c} ≥ 7%, and BMI ≥ 27 kg/m^2 was stratified by sex and by race/ethnicity. All risk scores were computed using the TIMI risk score algorithm. Error bars represent 95% CIs. ASCVD, atherosclerotic cardiovascular disease; BMI, body mass index; CI, confidence interval; CV, cardiovascular; DM, diabetes mellitus; HbA_{1c}, hemoglobin A_{1c}; LDL-C, low-density lipoprotein cholesterol; RRR, relative risk reduction.

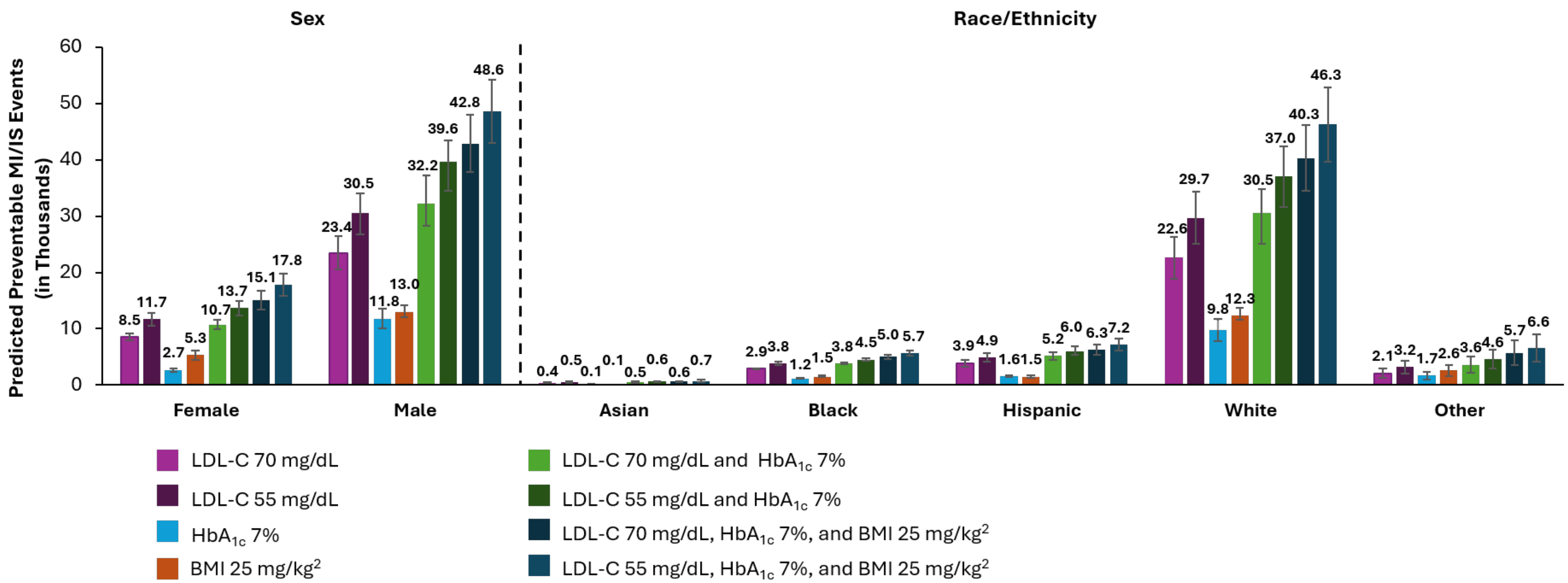

SUPPLEMENTAL FIGURE 4 Predicted Preventable CV Events With Added BMI Control by Sex and Race/Ethnicity

The number of predicted preventable MI and IS events over the course of 3 years that would result from control of LDL-C, HbA$_{1c}$, and BMI (individually and in combination) was estimated in the cohort with ASCVD, DM, LDL-C $\geq$ 70 mg/dL, HbA$_{1c}$ $\geq$ 7%, and BMI $\geq$ 27 kg/m^2 when stratified by sex and by race/ethnicity. All risk scores were computed using the TIMI risk score algorithm. Error bars represent 95% CIs. ASCVD, atherosclerotic cardiovascular disease; BMI, body mass index; CI, confidence interval; CV, cardiovascular; DM, diabetes mellitus; HbA$_{1c}$, hemoglobin A$_{1c}$; IS, ischemic stroke; LDL-C, low-density lipoprotein cholesterol; MI, myocardial infarction.
